# Supplementary material for: Clinical Utility of Patient-Derived Cell-Based In Vitro Drug Sensitivity Testing for Optimizing Adjuvant Therapy in Dogs with Solid Tumors: A Retrospective Study (2019–2023)
Source: Animals (Basel). 2025 Apr 16;15(8):1146. doi: 10.3390/ani15081146 (PMC12023965; doi:10.3390/ani15081146)
Supplement: Supplementary file 1 [file animals-15-01146-s001.zip › animals-3530077-supplementary.pdf]

Supplementary Material

Table S1. In vitro drug sensitivity testing results

Case no.	Dose-response curve	Selected drugs	Case no.	Dose-response curve	Selected drugs
1	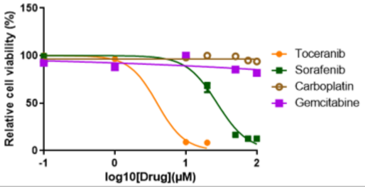	Toceranib	6	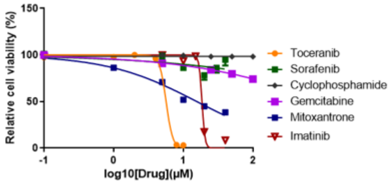	Toceranib
2	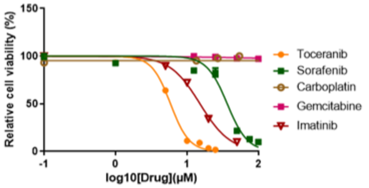	Toceranib	7	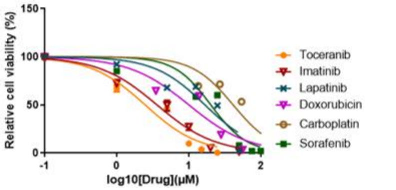	Toceranib
3	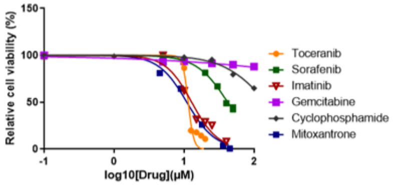	Toceranib	8	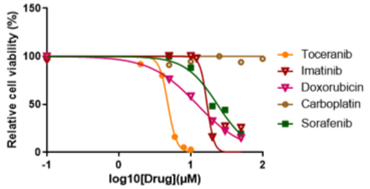	Toceranib
4	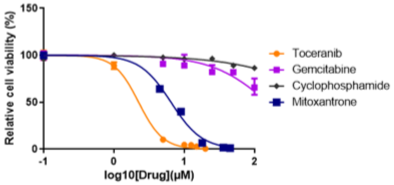	Toceranib	9	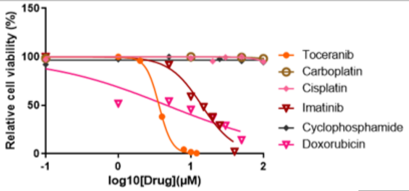	Toceranib
5	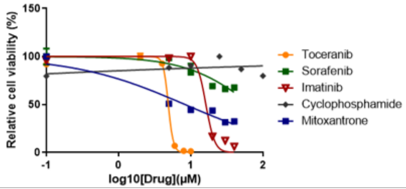	Toceranib	10	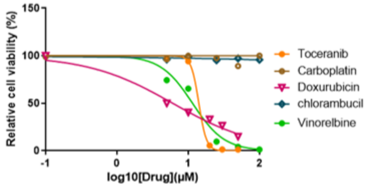	Doxorubicin
Case no.	Dose-response curve	Selected drugs	Case no.	Dose-response curve	Selected drugs

11	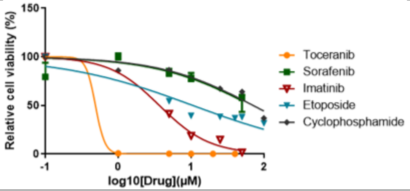	Toceranib	14	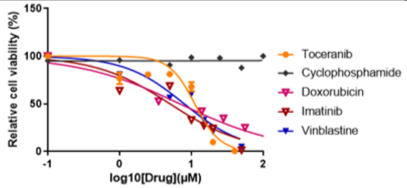	Imatinib
12	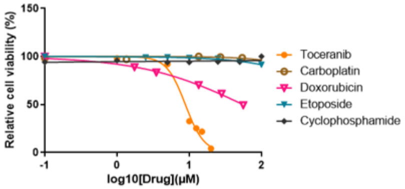	Doxorubicin	15	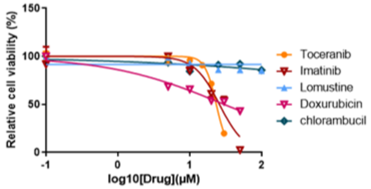	Toceranib
13	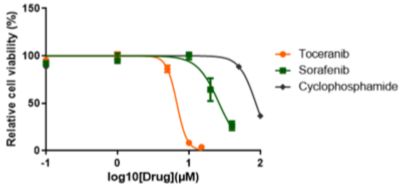	Toceranib	16	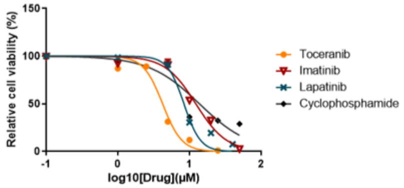	Toceranib

Table S2. Adverse events observed in dogs treated with TKIs and conventional chemotherapy

Category	Targeted therapy (n=26)					Conventional chemotherapy (n=7)				
	Grade 1	Grade 2	Grade 3	Grade 4	Grade 5	Grade 1	Grade 2	Grade 3	Grade 4	Grade 5
Constitutional										
Lethargy	5					5				
Gastrointestinal										
Inappetence	5	2				3	1			
Anorexia							1	1		
Vomiting	1	1				1	1	1		
Diarrhea	2	3					3	1		
Hematologic										
Anemia						1				
Neutropenia	6	1				1		1		
Thrombocytopenia										
Biochemical										
Increased ALT	2					1				
Increased AST	4									
Increased ALP	1								1	
Renal										
Proteinuria			1							
Cardiovascular										
Hypertension	1									
Total	27	7	1	0	0	12	6	4	1	0

Note: Adverse events were evaluated according to the Veterinary Cooperative Oncology Group-Common Terminology Criteria for Adverse Events (VCOG-CTCAE v2.0).

Abbreviation: ALT, alanine aminotransferase; AST, aspartate aminotransferase; ALP, alkaline phosphatase.
